# IGFBP3 Enhances Treatment Outcome and Predicts Favorable Prognosis in ABC-DLBCL

**DOI:** 10.1155/2023/1388041

**Published:** 2023-01-10

**Authors:** Han-Bing Li, Di Wang, Yue Zhang, Di Shen, Yi-Qun Che

**Affiliations:** ^1^Department of Clinical Laboratory, National Cancer Center, National Clinical Research Center for Cancer, Cancer Hospital, Chinese Academy of Medical Sciences and Peking Union Medical College, Beijing 100021, China; ^2^Department of Clinical Laboratory, Peking Union Medical College Hospital, Chinese Academy of Medical Sciences, Beijing 100730, China; ^3^Department of Clinical Laboratory, Beijing Chao-Yang Hospital, Capital Medical University, Beijing 100020, China; ^4^Center for Clinical Laboratory, Beijing Friendship Hospital, Capital Medical University, Beijing 100050, China

## Abstract

Chemoresistance is a key obstacle in the clinical treatment and management of activated B cell-like diffuse large B-cell lymphoma (ABC-DLBCL), which leads to the poor prognosis of patients. Exploring novel biomarkers to early warn drug resistance and ameliorate the patients' outcome in ABC-DLBCL is urgent and crucial. Previously, we found that insulin-like growth factor-binding protein 3 (IGFBP3) was remarkably associated with immunochemotherapy treatment response through microarray screening. Based on a retrospective cohort (*n* = 160) and a GEO cohort (*n* = 292), here we determined the positive expression rate of IGFBP3 and analyzed the role of IGFBP3 in treatment response and prognostics in ABC-DLBCL. The results demonstrated that the complete response (CR) rate of R-CHOP treatment was higher in ABC-DLBCL with IGFBP3 positive expression than those with IGFBP3 negative expression (42.0% vs 26.4%), and IGFBP3 positive expression in ABC-DLBCL was significantly correlated with enhanced therapeutic response (*P* = 0.037). High level of IGFBP3 was negatively correlated with tumorigenesis and development and predicted favorable survival time in ABC-DLBCL. In conclusion, IGFBP3 may be utilized as a promising biomarker for prognosis evaluation and a potential therapy target in ABC-DLBCL patients.

## 1. Introduction

The most common aggressive malignant tumor from the lymphatic system is diffuse large B-cell lymphoma (DLBCL), accounting for approximately 40%–50% of all non-Hodgkin lymphomas (NHL) [1]. Differences of genetic expression profiles indicated different B-cell differentiation stages, and the most widely used system identified molecularly distinct subtypes of DLBCL, the activated B cell-like (ABC) DLBCL, the germinal center B cell-like (GCB) DLBCL, and the unclassified [2]. Among Asian populations, especially in China, the ABC-DLBCL is the predominant subtype [3]. The emergence of high-flux gene sequencing techniques like full exome sequencing discovered the genetic pattern of DLBCL and thus improved the understanding of risk classification, therapeutic targets, and prognostic evaluation [4]. The prognosis of patients in the ABC subtype is inferior to that of the GCB subtype, and the heterogeneity can be traced to NOTCH1, MYD88, and CD79B mutations [5]. Even though they share the same immunohistochemical subtype, the risk and prognosis are still different. In the MCD and N1 gene subtypes, the 3-year event-free survival was higher in patients ≤60 years of age treated with R-CHOP in combination with ibrutinib, whereas the prognosis of patients in BN2 was much poorer [6]. In that case, more novel molecules are needed to improve the diagnosis during the management of DLBCL. Currently, rituximab plus cyclophosphamide, doxorubicin, vincristine, and prednisone (R-CHOP) is the front-line standard treatment for DLBCL. Despite the fact that about 60% of newly-diagnosed DLBCL cases can be cured by this therapy, up to 45%–50% of patients will relapse or become refractory [7]. The survival of patients with refractory DLBCL was poor, with a median overall survival of 6.3 months and 20% of 2-year survival [8]. Therefore, exploring novel therapeutic targets to increase the complete response (CR) rate and screening for biomarkers to warn chemotherapy resistance and further to improve the prognostics of DLBCL patients are of clinical value.

On the basis of the previous work of our research group and literature searching, we found that insulin-like growth factor-binding protein 3 (IGFBP3) was markedly associated with the prognosis of ABC-DLBCL. IGFBP3, a member of the IGFBP family, plays a major role as a carrier of IGFs in circulation and regulates the availability and activity of IGF [9]. IGFBP3 has been proven to exhibit an anti-tumor effect in a p53-dependent manner, and the down-regulation of IGFBP3 implicates with neuroendocrine differentiation, migration and invasion, and therapeutic sensitivity in various tumors [10–12]. IGFBP3 overexpression reduced the cell metastasis of head and neck squamous cell carcinoma (HNSCC), while the silence of IGFBP3 enhanced the metastatic phenotype in vitro and in vivo [13]. Besides, IGFBP3 re-sensitized chemo-resistant pancreatic ductal adenocarcinoma cells by activating apoptosis through Bcl-2 downregulation, Bax upregulation, caspase 3 and caspase 8 activation [14]. Moreover, higher level of IGFBP3 expression was predictive of superior survival in patients with hepatocellular carcinoma and may be an independent prognostic biomarker [15]. However, the relationship between IGFBP3 and ABC-DLBCL has not been reported so far.

Given the fact that IGFBP3 suppression contributed to tumorigenesis and progression, here we determined the positive expression rate of IGFBP3, analyzed the role of IGFBP3 in treatment response and prognostic assessment of ABC-DLBCL, and further validated our results using the GEO database.

## 2. Materials and Methods

### 2.1. Patients

Tumor tissues of 160 patients with newly diagnosed ABC-DLBCL were collected at the National Cancer Center/Cancer Hospital, Chinese Academy of Medical Sciences (Beijing, China). The inclusion criteria of the patients were as follows: ① pathological diagnosis of ABC-DLBCL; ② R-CHOP treatment for 6–8 cycles; ③ no heart, liver, kidney, or other diseases; ④ complete clinical records. The exclusion criteria were as follows: ① primary mediastinal large B-cell lymphoma; ② primary DLBCL in the testis or central nervous system. All patients enrolled were provided with written informed consent in advance. The study was approved by Cancer Hospital, Chinese Academy of Medical Sciences ethics committee, and the procedures were carried out in accordance with the Declaration of Helsinki.

### 2.2. Tissue Microarray Preparation

The tumor tissues collected were stored at −80°C. After being removed from the ultra-low temperature freezer, they were fixed with formalin and then embedded with paraffin wax. The tissues were then cut into 4 *μ*m-thick sections and stained with HE to label the tumor area under the microscope. Then, the tumor areas were prepared into wax blocks and re-embedded. Two spots of each tumor tissue were taken, and the prepared wax blocks were cut into 5 *μ*m/piece chips for subsequent experiments.

### 2.3. Detection of IGFBP3 Using RNA In Situ Hybridization

RNA in situ hybridization (RNAscope) was utilized for microarray analysis to determine the expression level of IGFBP3 in ABC-DLBCL according to the previously published method [16]. The human gene IGFBP3 probe was provided by Advanced Cell Diagnostics (ACD, Newark).

### 2.4. Microarray Data Preprocessing

Clinical data and gene expression profiles of 292 ABC-DLBCL patients were retrieved from the GSE10846 and GSE31312 datasets in the Gene Expression Omnibus (GEO) database (platform GPL570). The inclusion criteria of patients were as follows: ① pathological diagnosis was ABC-DLBCL; ② all received 6–8 cycles of R-CHOP treatment; ③ being without other malignant tumors. The raw data from the GEO database were normalized by the “rma” and “mas5” algorithm using “affy” package in R software. The batch effect was eliminated by the “normalizeBetweenArrays” and “removeBatchEffect” algorithm using the “limma” package. Then, the logarithmic transformation was used to convenient subsequent analyses.

### 2.5. Data Statistics

The SPSS 26.0 software (IBM, USA) was used for statistical analysis, and all figures were drawn by GraphPad Prism 8.0 (Graph Pad Software, USA). The Chi-square test was applied to compare the correlation between IGFBP3 expression and clinicopathological characteristics. The role of IGFBP3 in overall survival (OS) and progression-free survival (PFS) of patients with ABC-DLBCL was then assessed using the log-rank (Manteler–Cox) test, and Kaplan–Meier survival curves were plotted based on prognostic data. Next, univariate and multivariate Cox regression analyses were performed for OS and PFS to determine the independent prognostic features of ABC-DLBCL. The variables of *p* values <0.15 in the univariate analysis were brought into the multivariate analysis. All statistical tests were two-sided, and *p* < 0.05 was considered statistically significant.

## 3. Results

### 3.1. CR Rate of R-CHOP Treatment Was Higher in ABC-DLBCL with IGFBP3 Positive Expression

**Figure 1:**
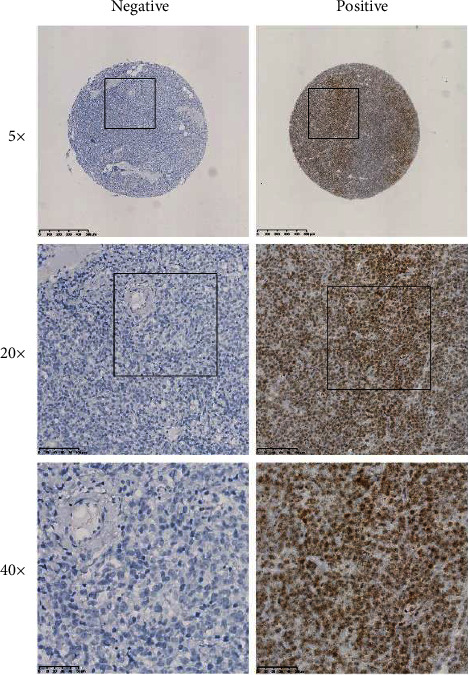
Tissue images of RNA in situ hybridization for IGFBP3 differential expression in ABC-DLBCL.

A total of 160 newly diagnosed ABC-DLBCL cases treated with R-CHOP were enrolled retrospectively. The clinicopathological factors of ABC-DLBCL patients including age, sex, stage, extranodal infiltration, ECOG score, LDH, IPI score, and treatment response are listed in Table 1. RNA in situ hybridization was operated on tissue samples to determine the expression level of IGFBP3. As was shown, the positive rate of IGFBP3 mRNA expression was 46.2% (36/78) in stage I-II patients and 40.2% (33/82) in stage III-IV patients (Figure 1). The CR rate of R-CHOP treatment was higher in ABC-DLBCL with IGFBP3 positive expression than those with IGFBP3 negative expression (42.0% vs 26.4%). The positive expression of IGFBP3 in ABC-DLBCL was remarkably connected with a favorable therapeutic response (*P* = 0.037) (Table 1).

### 3.2. High IGFBP3 Expression Was Negatively Correlated with Advanced Clinical Characteristics in ABC-DLBCL Patients in the GEO Database

In order to further clarify the role of IGFBP3 in regulating the development of ABC-DLBCL, a total of 292 cases were retrieved from the GEO database for subsequent analysis. The lower 25% of IGFBP3 level was identified as a low expression subset and the higher 25% as the high expression subset. The results revealed that low expression of IGFBP3 was closely correlated with advanced tumor stage (*P* = 0.020), involvement of >1 extranodal sites (*P* = 0.033), higher IPI score (*P* = 0.007), and elevated LDH levels (*P* = 0.011) (Table 2). Nevertheless, the features of age, sex, ECOG score and treatment response exhibited no statistically differences. These evidences demonstrated that IGFBP3 involved in negatively regulating the clinical development of ABC-DLBCL.

### 3.3. High IGFBP3 Expression Predicted Superior Prognosis in ABC-DLBCL Patients

**Figure 2:**
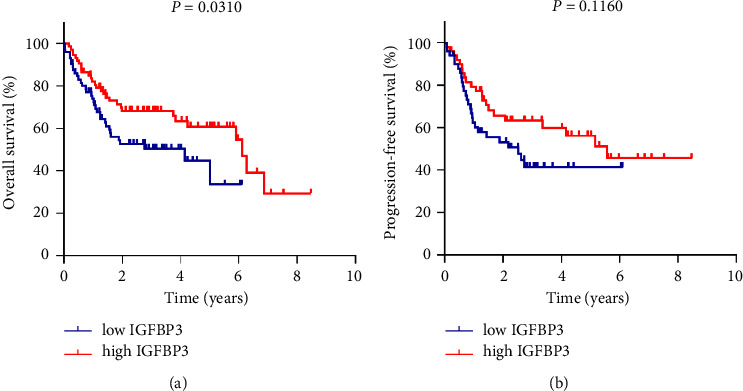
Kaplan–Meier survival curves for OS and PFS of patients with ABC-DLBCL in different IGFBP3 expression levels from the GEO database.

Next, we investigated the prognostic value of IGFBP3 expression level in ABC-DLBCL patients from the GEO cohort through log-rank test. The analysis indicated that OS of ABC-DLBCL patients with IGFBP3 high expression was superior to those with IGFBP3 low expression (*P* = 0.0310) (Figure 2(a)). The PFS of the IGFBP3 highexpression group had a trend to be better than that of the lowexpression group, though there was no statistical significance (*P* = 0.1160) (Figure 2(b)).

### 3.4. IGFBP3 Tended to Be an Independent Prognostic Biomarker for PFS of ABC-DLBCL

Furthermore, univariate and multivariate Cox regression analyses were carried out in order to analyze whether IGFBP3 independently effected the prognosis of ABC-DLBCL. The results exhibited that IGFBP3 level, age, stage, extranodal infiltration, ECOG score, LDH, IPI score, and treatment response were clinical characteristics associated with OS in the univariate analysis (Table 3). ECOG score was an independent characteristic for OS in the multivariate analysis (*P* = 0.008, HR: 2.952, 95% CI: 1.334–6.531). In terms of PFS, IGFBP3 level, stage, extranodal infiltration, ECOG score, LDH, and IPI score involved in effecting the prognosis (Table 4). Multivariate Cox regression analysis demonstrated that IGFBP3 tended to be an independent prognostic element for PFS (*P* = 0.055, HR: 0.503, 95% CI: 0.249–1.016) and LDH independently predicted the prognosis of ABC-DLBCL (*P* = 0.017, HR: 1.348, 95% CI: 0.145–0.831).

## 4. Discussion

R-CHOP immunochemotherapy has been the standard first-line regimen for patients with DLBCL for decades. However, relapse and refractory of DLBCL are key problems in clinical practice, which remarkably affect the outcome of DLBCL patients. For recurrent and refractory DLBCL patients, chemo-resistance is the main obstacle remained to be solved. Therefore, exploring novel tumor biomarkers to early warn drug resistance and developing potential targets to improve treatment efficiency have important clinical implications. Proteomics and transcriptomics research has found some molecules that are related to the immunotherapy and chemotherapy response in DLBCL. Nuclear protein CYCLON and nucleolar multifunctional protein NPM1 were found to be associated with DLBCL advance and anti-CD20 therapy resistance, which may be potential novel targets for DLBCL treatment [17]. Besides, miR-197 low expression was significantly correlated with progression and prognosis of DLBCL and played a chemo-sensitizing effect in ABC-DLBCL [18]. However, metabolism-related studies in DLBCL treatment resistance were relatively few compared with proteomics and transcriptomics studies.

The molecular landscape of cancer metabolism and tumor microenvironment opens up a new perspective for understanding the characteristics of DLBCL. The insulin-like growth factors (IGFs) and the family of IGFBPs involve in metabolism regulation and sustain metabolic homeostasis. Among them, IGFBP3 is the most plentiful circulating IGFBP and carries out the main IGF transport function [19]. IGFBP3 is an essential molecule that controls cellular activities such as cell proliferation, differentiation, and apoptosis. Previous studies had demonstrated that the absence of IGFBP3 was involved in intrinsic or acquired resistance to chemotherapy or targeting therapy in different types of cancer. IGFBP3 expression was significantly down-regulated in cisplatin-resistant non-small cell lung cancer (NSCLC) cells, and overexpression of IGFBP3 enhanced the cisplatin response by blocking IGF1 signaling [20]. IGFBP3 expression was down-regulated in Her2-positive breast cancer cells with trastuzumab resistance and led to the inhibition of the Wnt pathway and the rise of Cullin7 expression mediated by TCF7L2 [21]. Excessive expression of IGFBP3 dominantly repressed cell growth in prostate cancer, and the enhancement of docetaxel cytotoxicity in prostate cancer cells by low-dose dihydrotestosterone/calcitriol depended on IGFBP3 expression [22]. Nevertheless, the role of IGFBP3 in ABC-DLBCL has not been documented up to now. These discoveries inspired us to explore the relationship between the metabolism-related molecule IGFBP3 and R-CHOP treatment response in patients with ABC-DLBCL.

The present study initially identified the expression of IGFBP3 in ABC-DLBCL tissues using RNAscope. RNAscope makes rapid molecular diagnostic assays of tumor biomarkers based on RNA in situ hybridization (RISH) with high sensitivity and specificity. The results demonstrated that 46.2% (36/78) in stage I-II and 40.2% (33/82) in stage III-IV of ABC-DLBCL cases were positive for IGFBP3 mRNA expression. More importantly, compared with IGFBP3 negative expression patients, the CR rate of R-CHOP treatment was obviously better in those with IGFBP3 positive expression (42.0% vs 26.4%). The positive level of IGFBP3 in ABC-DLBCL was significantly correlated with a higher R-CHOP treatment response (*P* = 0.037).

Providing IGFBP3 expression influenced the therapeutic effect, we further clarified how IGFBP3 regulated ABC-DLBCL tumorigenesis and development. The Chi-square analysis exhibited that high IGFBP3 was negatively correlated with tumor stage, extranodal infiltration, LDH levels, and IPI score, which demonstrated IGFBP3 involved in repressing ABC-DLBCL clinicopathological progression. The revised-IPI(R-IPI) has shown excellent performance in risk stratification and prognostic evaluation of DLBCL patients. The patients with very high-risk R-IPI have 100% 3-year PFS (95%CI: 88%-100%), and R-IPI predicted outcome with high accuracy according to a Danish-Canadian study consisting of 443 patients diagnosed with DLBCL [23]. It is worth noting that the treatment response in the GEO cohort did not exhibit a statistical difference. The main reason may be the different CR rates due to the different patients enrolled. Patients in GSE10846 were from the Lymphoid Malignancies Branch, National Cancer Institute, USA, and patients in GSE31312 were from the Department of Bioinformatics, Roche Molecular Systems. In that case, patients in different regions may have discrepancies in race and treatment responsiveness, reflecting by the fact that the CR rate in the GEO cohort was obviously higher than our cohort (74.0% vs 33.1%).

Since IGFBP3 conveyed a suppression on ABC-DLBCL development and a negative correlation with IPI score, the relationship between IGFBP3 and prognosis of ABC-DLBCL was analyzed. The results exhibited that the OS of ABC-DLBCL patients with IGFBP3 high expression was favorable and the PFS of the IGFBP3 high expression group had a trend to be better compared with those with IGFBP3 low expression. Univariate and multivariate Cox regression statistics indicated that IGFBP3 tended to be an independent prognostic biomarker for PFS of ABC-DLBCL. High expression of IGFBP3 was presented as a protective role and may serve as a promising signal in the prognostic assessment of patients with ABC-DLBCL. IGFBP3 has shown its value in the prognosis evaluation of different tumors. The levels of IGFBP3 mRNA and protein were decreased in pancreatic ductal adenocarcinoma, and IGFBP3 was positively associated with OS [24]. In addition, a high IGFBP3 level was significantly associated with an early, nonserous histology and optimal cell reduction of invasive epithelial ovarian cancer, and the OS and PFS times were significantly better among patients with high IGFBP3 levels [25]. However, an observational and Mendelian randomization analysis from the UK Biobank found that IGFBP3 concentrations were not related to the risk of breast cancer (OR = 1.00) [26]. In addition, another serologic analysis based on a genome-wide association study revealed that the level of IGFBP3 predicted an increasing risk of colorectal cancer (OR = 1.12) [27]. These inconsistencies enlighten researchers to comprehensively consider the risk assessment of IGFBP3 according to specific tumors.

Although IGFBP3 performed well in the prognosis monitoring of ABC-DLBCL, certain limitations in our study should be taken into consideration. First of all, the PFS of our cohort did not exhibit a significant difference, which may be due to the bias occurring in judging tumor progression. Further trace and record are needed to provide more complete prognostic analysis. Besides, contribution to or suppression on carcinogenesis depends on the molecular mechanism IGFBP3 involves, thus biological experiments in vivo and vitro are required to determine the signaling pathway.

In conclusion, ABC-DLBCL patients with IGFBP3 positive expression had a stronger CR rate with R-CHOP treatment. Elevated level of IGFBP3 was negatively associated with ABC-DLBCL clinical development. IGFBP3 may be a promising biomarker to predict a favorable prognosis of ABC-DLBCL and a potential target for ABC-DLBCL precision medicine in clinical practice.

## Figures and Tables

**Table 1:** Correlations between the expression level of IGFBP3 and clinicopathological factors of ABC-DLBCL patients.

Variables		Expression level of IGFBP3		*p* value
		Negative	Positive	
Age				0.472
	>60	34	22	
	≤60	57	47	

Sex				0.395
	Female	40	35	
	Male	51	34	

Stage				0.451
	I-II	42	36	
	III-IV	49	33	

Extranodal infiltration				0.301
	≤1	52	45	
	>1	39	24	

ECOG score				0.383
	≤1	73	59	
	>1	18	10	

LDH				0.943
	Normal	48	36	
	Elevated	43	33	

IPI score				0.383
	≤2	76	61	
	>2	15	8	

Treatment response				**0.037**
	CR	24	29	
	Non-CR	67	40	

ECOG, eastern cooperative oncology group performance status; LDH, lactate dehydrogenase; IPI, international prognostic index; CR, complete response; non-CR, partial response, stable disease, progressive disease; bold, significant difference.

**Table 2:** Correlations between the expression level of IGFBP3 and clinicopathological factors in ABC-DLBCL patients from the GEO database.

Variables		Expression level of IGFBP3		*p* value
		Low	High	
Age				0.159
	≤60	20	28	
	>60	53	45	

Sex				0.238
	Female	33	26	
	Male	40	47	

Stage				**0.020**
	I-II	25	39	
	III-IV	48	34	

Extranodal infiltration				**0.033**
	≤1	50	61	
	>1	23	12	

ECOG score				0.442
	≤1	53	57	
	>1	20	16	

LDH				**0.011**
	Normal	22	37	
	Elevated	51	36	

IPI score				**0.007**
	≤2	36	52	
	>2	37	21	

Treatment response				0.706
	CR	53	55	
	Non-CR	20	18	

ECOG, eastern cooperative oncology group performance status; LDH, lactate dehydrogenase; IPI, international prognostic index; CR, complete response; non-CR, partial response, stable disease, progressive disease; bold, significant difference.

**Table 3:** Univariate and multivariate Cox regression analysis of OS from GEO.

Variables	Univariate Cox			Multivariate Cox		
	HR	95% CI	*p*	HR	95% CI	*p*
IGFBP3 expression (high vs low)	0.604	0.365–0.997	**0.049**	0.722	0.367–1.419	0.345
Age (>60 vs ≤60)	1.588	1.067–2.363	**0.023**	1.508	0.664–3.427	0.327
Sex (male vs female)	0.869	0.609–1.240	0.439			
Stage (III + IV vs I + II)	2.019	1.365–2.986	**<0.001**	1.778	0.329–1.837	0.567
Extranodal infiltration (>1 vs ≤1)	2.191	1.485–3.234	**<0.001**	1.069	0.327–3.500	0.912
ECOG score (>1 vs ≤1)	2.779	1.901–4.062	**<0.001**	2.952	1.334–6.531	**0.008**
LDH (elevated vs normal)	1.955	1.264–3.024	**0.003**	1.837	0.298–2.354	0.736
IPI score (>2 vs ≤2)	3.615	2.168–6.026	**<0.001**	2.368	0.606–9.261	0.215
Treatment response (non-CR vs CR)	1.581	0.953–2.624	**0.076**	1.309	0.522–3.283	0.566

ECOG score, eastern cooperative oncology group score; LDH, lactate dehydrogenase; CR, complete response; non-CR, partial response, stable disease, progressive disease; bold, significant difference.

**Table 4:** Univariate and multivariate Cox regression analysis of PFS from GEO.

Variables	Univariate Cox			Multivariate Cox		
	HR	95% CI	*p*	HR	95% CI	*p*
IGFBP3 expression (high vs low)	0.624	0.350–1.112	**0.110**	0.503	0.249–1.016	**0.055**
Age (>60 vs ≤60)	1.167	0.754–1.806	0.489			
Sex (male vs female)	0.822	0.544–1.240	0.349			
Stage (III + IV vs I + II)	2.372	1.503–3.744	**<0.001**	1.005	0.420–2.407	0.990
Extranodal infiltration (>1 vs ≤1)	2.314	1.495–3.582	**<0.001**	1.332	0.648–2.737	0.435
ECOG score (>1 vs ≤1)	2.252	1.429–3.549	**<0.001**	1.609	0.723–3.582	0.244
LDH (elevated vs normal)	1.482	0.918–2.390	**0.107**	1.348	0.145–0.831	**0.017**
IPI score (>2 vs ≤2)	3.209	1.840–5.598	**<0.001**	2.914	0.850–9.985	0.089
Treatment response (non-CR vs CR)	1.106	0.616–1.988	0.735			

ECOG score, eastern cooperative oncology group score; LDH, lactate dehydrogenase; CR, complete response; non-CR, partial response, stable disease, progressive disease; bold, significant difference.

## Data Availability

All data analyzed in this study are included in the article. Raw data and material will be available from the corresponding author on reasonable request.

## References

[1] Bowzyk Al-Naeeb A., Ajithkumar T., Behan S., Hodson D. J. (2018). Non-Hodgkin lymphoma. *BMJ*.

[2] Alizadeh A. A., Eisen M. B., Davis R. E. (2000). Distinct types of diffuse large B-cell lymphoma identified by gene expression profiling. *Nature*.

[3] Chen Y., Dave B. J., Zhu X. (2013). Differences in the cytogenetic alteration profiles of diffuse large B-cell lymphoma among Chinese and American patients. *Cancer Genetics*.

[4] Miao Y., Medeiros L. J., Li Y., Li J., Young K. H. (2019). Genetic alterations and their clinical implications in DLBCL. *Nature Reviews Clinical Oncology*.

[5] Schmitz R., Wright G. W., Huang D. W. (2018). Genetics and pathogenesis of diffuse large B-cell lymphoma. *New England Journal of Medicine*.

[6] Wilson W. H., Wright G. W., Huang D. W. (2021). Effect of ibrutinib with R-CHOP chemotherapy in genetic subtypes of DLBCL. *Cancer Cell*.

[7] Susanibar-Adaniya S., Barta S. K. (2021). 2021 Update on Diffuse large B cell lymphoma: a review of current data and potential applications on risk stratification and management. *American Journal of Hematology*.

[8] Crump M., Neelapu S. S., Farooq U. (2017). Outcomes in refractory diffuse large B-cell lymphoma: results from the international SCHOLAR-1 study. *Blood*.

[9] Cai Q., Dozmorov M., Oh Y. (2020). IGFBP-3/IGFBP-3 receptor system as an anti-tumor and anti-metastatic signaling in cancer. *Cells*.

[10] Mayo J. C., Hevia D., Quiros-Gonzalez I. (2017). IGFBP3 and MAPK/ERK signaling mediates melatonin-induced antitumor activity in prostate cancer. *Journal of Pineal Research*.

[11] Fan X., Wang Y., Jiang T. (2018). B-myb mediates proliferation and migration of non-small-cell lung cancer via suppressing IGFBP3. *International Journal of Molecular Sciences*.

[12] Chen L., Alexe G., Dharia N. V. (2017). CRISPR-Cas9 screen reveals a MYCN-amplified neuroblastoma dependency on EZH2. *Journal of Clinical Investigation*.

[13] Le H. T., Lee H. J., Cho J. (2021). Insulin-like growth factor binding protein-3 exerts its anti-metastatic effect in aerodigestive tract cancers by disrupting the protein stability of vimentin. *Cancers*.

[14] Mofid M. R., Gheysarzadeh A., Bakhtiyari S. (2020). Insulin-like growth factor binding protein 3 chemosensitizes pancreatic ductal adenocarcinoma through its death receptor. *Pancreatology*.

[15] Yan J., Yang X., Li L. (2017). Low expression levels of insulin-like growth factor binding protein-3 are correlated with poor prognosis for patients with hepatocellular carcinoma. *Oncology Letters*.

[16] Wang D., Zhang Y., Che Y. Q. (2020). CCND2 mRNA expression is correlated with R-CHOP treatment efficacy and prognosis in patients with ABC-DLBCL. *Frontiers in Oncology*.

[17] Bouroumeau A., Bussot L., Hamaidia S. (2021). CYCLON and NPM1 cooperate within an oncogenic network predictive of R-CHOP response in DLBCL. *Cancers*.

[18] Yang J. M., Jang J. Y., Jeon Y. K., Paik J. H. (2018). Clinicopathologic implication of microRNA-197 in diffuse large B cell lymphoma. *Journal of Translational Medicine*.

[19] Haywood N. J., Slater T. A., Matthews C. J., Wheatcroft S. B. (2019). The insulin like growth factor and binding protein family: novel therapeutic targets in obesity & diabetes. *Molecular Metabolism*.

[20] Wang Y. A., Sun Y., Palmer J. (2017). IGFBP3 modulates lung tumorigenesis and cell growth through IGF1 signaling. *Molecular Cancer Research*.

[21] Qiu N., He Y. F., Zhang S. M. (2019). Cullin7 enhances resistance to trastuzumab therapy in Her2 positive breast cancer via degrading IRS-1 and downregulating IGFBP-3 to activate the PI3K/AKT pathway. *Cancer Letters*.

[22] Igarashi K., Yui Y., Watanabe K. (2020). Molecular evidence of IGFBP-3 dependent and independent VD3 action and its nonlinear response on IGFBP-3 induction in prostate cancer cells. *BMC Cancer*.

[23] El-Galaly T. C., Villa D., Alzahrani M. (2015). Outcome prediction by extranodal involvement, IPI, R-IPI, and NCCN-IPI in the PET/CT and rituximab era: a Danish-Canadian study of 443 patients with diffuse-largeB-cell lymphoma. *American Journal of Hematology*.

[24] Gheysarzadeh A., Bakhtiari H., Ansari A., Emami Razavi A., Emami M. H., Mofid M. R. (2019). The insulin-like growth factor binding protein-3 and its death receptor in pancreatic ductal adenocarcinoma poor prognosis. *Journal of Cellular Physiology*.

[25] Huang Y. F., Cheng W. F., Wu Y. P., Cheng Y. M., Hsu K. F., Chou C. Y. (2014). Circulating IGF system and treatment outcome in epithelial ovarian cancer. *Endocrine-Related Cancer*.

[26] Murphy N., Knuppel A., Papadimitriou N. (2020). Insulin-like growth factor-1, insulin-like growth factor-bindingprotein-3, and breast cancer risk: observational and Mendelian randomization analyses with ∼430 000 women. *Annals of Oncology*.

[27] Murphy N., Carreras-Torres R., Song M. (2020). Circulating levels of insulin-like growth factor 1 and insulin-like growth factor binding protein 3 associate with risk of colorectal cancer based on serologic and mendelian randomization analyses. *Gastroenterology*.

